# Supplementary material for: Reducing Supply Chain Dependencies for Viral Genomic Surveillance: Get by with a Little HELP from Commercial Enzymes already in your Lab Freezer
Source: Wellcome Open Res. 2026 Apr 21;10:529. Originally published 2025 Oct 1. [Version 2] doi: 10.12688/wellcomeopenres.24735.2 (PMC13197761; doi:10.12688/wellcomeopenres.24735.2)
Supplement: Peer review history

# Reviewer report: Reviewer response for version 2

**DOI:** 10.21956/wellcomeopenres.29150.r155591

**Published:** 2026-05-22

**Authors:** Aloysious Ssemaganda

**Affiliations:** 1 Central Public Health Laboratories, Kampala, Uganda

This manuscript presents a highly relevant, reproducible, and cost-effective workflow for viral genomic surveillance. The methodology is technically sound and presented with excellent clarity. However, conclusions regarding Norovirus GII demand cautious interpretation; the assay’s performance was significantly constrained by genotype-specific primer-scheme limitations rather than the inherent capabilities of the proposed protocol. Further optimization remains necessary for broader clinical diagnostic applications.

Is the work clearly and accurately presented and does it cite the current literature?

Yes

If applicable, is the statistical analysis and its interpretation appropriate?

I cannot comment. A qualified statistician is required.

Are all the source data underlying the results available to ensure full reproducibility?

Yes

Is the study design appropriate and is the work technically sound?

Yes

Are the conclusions drawn adequately supported by the results?

Yes

Are sufficient details of methods and analysis provided to allow replication by others?

Yes

Reviewer Expertise:

I am an Infectious Disease Scientist dedicated to translating molecular research into tangible public health solutions. My work focuses on the design and execution of clinical studies, with a specialization in immunology and molecular epidemiology aimed at addressing the world's most challenging high-burden pathogens

I confirm that I have read this submission and believe that I have an appropriate level of expertise to confirm that it is of an acceptable scientific standard.

# Reviewer report: Reviewer response for version 1

**DOI:** 10.21956/wellcomeopenres.27252.r141594

**Published:** 2025-12-19

**Authors:** Tibutius Thanesh Pramanayagam Jayadas

**Affiliations:** 1 University of Sri Jayewardenepura, Nugegoda, Sri Lanka

**Are sufficient details of methods and analysis provided to allow replication by others?**

 Partly

 Core conditions are described (RT temperatures, PCR cycling, library prep conceptual composition, sequencing and bioinformatics overview).  The authors also provide extended data and protocols via GitHub/protocols.io, which is positive. 

 However, for replication from the manuscript alone, several critical implementation details are either externalized or not fully specified: Replication-critical details to bring into the paper (must address):

 Replicates and randomization: For each workflow/condition state the number of independent replicates and whether comparisons were run on the same flow cell or across multiple runs (this strongly affects depth and dropout comparisons). 

 Input normalization: Clarify whether amplicon inputs into EP/BL/AL were normalized to the same ng amount across workflows and samples (you mention quantification specify normalization strategy). 

 Exact barcoding/ligation stoichiometry: You describe barcode dilution and volumes; add the final molar or mass assumptions.

 Computational reproducibility: Provide versions/commit hashes for ARTIC pipeline, medaka model version, ncov-tools version and basecalling model details beyond “high accuracy model,” because these change consensus outcomes. 

**If applicable, is the statistical analysis and its interpretation appropriate?**

 Partly

 For a methods/workflow paper, descriptive metrics and visualizations are acceptable.   However, the manuscript repeatedly compares workflows (sometimes with small differences) without quantifying uncertainty, effect sizes or reproducibility. There are also multiple conditions (polymerases, mixes, Ct strata) which is exactly where minimal statistics would strengthen interpretability.

**Are the conclusions drawn adequately supported by the results?**

 Partly

 The central operational conclusion HELP can achieve ARTIC-like coverage for many samples, especially at lower Ct / higher viral load and can maintain sequencing continuity during shortages is supported by the presented coverage and dropout data. 

Where conclusions drift beyond the data:

 Claims about general robustness may be too broad given limited external validation (single lab setting, limited number of enzymes tested, limited replication described). 

 The cost inequity discussion is important but the inference that disparities are driven “rather than protocol complexity” is not fully demonstrated complexity is not quantitatively measured and procurement ecosystems differ. The table shows price variation the causal attribution should be softened or better supported. 

 Norovirus conclusions should be framed carefully: the data strongly suggest genotype/primer-scheme effects dominate coverage for some genotypes but additional experiments (primer mismatch analysis, rebalancing, alternate schemes) would be needed before recommending HELP broadly for “Norovirus GII” without caveats.

Is the work clearly and accurately presented and does it cite the current literature?

Yes

If applicable, is the statistical analysis and its interpretation appropriate?

Partly

Are all the source data underlying the results available to ensure full reproducibility?

Yes

Is the study design appropriate and is the work technically sound?

Yes

Are the conclusions drawn adequately supported by the results?

Partly

Are sufficient details of methods and analysis provided to allow replication by others?

Partly

Reviewer Expertise:

Molecular Biology, Virology, Infectious diseases

I confirm that I have read this submission and believe that I have an appropriate level of expertise to confirm that it is of an acceptable scientific standard, however I have significant reservations, as outlined above.

# Response

**Published:** 2026-04-11

**Authors:** Ganna Kovalenko

**Affiliations:** University of California Irvine, Irvine, California, USA

We sincerely thank the reviewer for the thoughtful and constructive feedback, which helped us improve the clarity of the manuscript.

**Reviewer Comment 1:**  Core conditions are described (RT temperatures, PCR cycling, library prep conceptual composition, sequencing and bioinformatics overview).  The authors also provide extended data and protocols via GitHub/protocols.io, which is positive. However, for replication from the manuscript alone, several critical implementation details are either externalized or not fully specified: Replication-critical details to bring into the paper (must address):

**1)** Replicates and randomization: For each workflow/condition state the number of independent replicates and whether comparisons were run on the same flow cell or across multiple runs (this strongly affects depth and dropout comparisons). 

**Response: **We thank the reviewer for these helpful comments and for highlighting important points to improve our study. The experimental design was organised into three sequential stages, each performed on a separate flow cell. Within each stage, workflows were evaluated by preparing separate libraries from the same amplicon pools, enabling direct comparison of workflows under the same experimental conditions. Libraries prepared with different adapter ligation (AL) mixes were sequenced on separate flow cells, as Oxford Nanopore library preparation protocols require libraries loaded on the same flow cell to use the same adapter mix. **We have now clarified this in the Methods section:** *(lines 123-125): Within each stage, workflows were compared by preparing separate libraries from the same amplicon pools under identical experimental conditions.* *(lines 175-177): Experiments were organised into sequential stages, with each stage sequenced on a new flow cell. Libraries prepared with different adapter ligation mixes were sequenced on separate flow cells following ONT library preparation requirements.*

**2)** Input normalization: Clarify whether amplicon inputs into EP/BL/AL were normalized to the same ng amount across workflows and samples (you mention quantification specify normalization strategy). Exact barcoding/ligation stoichiometry: You describe barcode dilution and volumes; add the final molar or mass assumptions.

**Response: **We appreciate the reviewer's helpful suggestions regarding input normalization and reaction stoichiometry. Amplicon pools were quantified after PCR cleanup as a quality control step to assess amplicon yield and suitability for library preparation. However, library preparation followed the ARTIC LoCost workflow, which uses fixed reaction volumes rather than normalization to a defined DNA mass or molar input. As HELP was designed as a drop-in alternative to the ARTIC LoCost protocol, we followed the same volume-based reaction setup for end-prep, barcoding, and adapter ligation steps. This approach is widely used and has been extensively validated in nanopore-based viral genome sequencing **.**

**We have now clarified this in the Methods section**: *(lines 125–127): End-prep, barcoding and adapter ligation reactions were performed using fixed reaction volumes according to the ARTIC LoCost protocol rather than molar normalization of DNA input.*

**3)** Computational reproducibility: Provide versions/commit hashes for ARTIC pipeline, medaka model version, ncov-tools version and basecalling model details beyond “high accuracy model,” because these change consensus outcomes. 

**Response: **We appreciate the reviewer's comments. To improve computational reproducibility, we have now specified the versions of the ARTIC pipeline, medaka, and ncov-tools ( *lines 179-185*), as well as the basecalling model configuration (dna_r9.4.1_e8_hac@v3.3) used ( *line 175*). These details have been added to the Methods section of the revised manuscript.

**Reviewer Comment 2:** **If applicable, is the statistical analysis and its interpretation appropriate?**

 For a methods/workflow paper, descriptive metrics and visualisations are acceptable.   However, the manuscript repeatedly compares workflows (sometimes with small differences) without quantifying uncertainty, effect sizes or reproducibility. There are also multiple conditions (polymerases, mixes, Ct strata) which is exactly where minimal statistics would strengthen interpretability.

**Response:** We appreciate this important suggestion. We have revised the Results section to strengthen interpretability by incorporating quantitative comparisons across workflows and conditions. Specifically, we now report genome coverage ranges across Ct strata in Stages 1-3 and refine comparisons across polymerases and library preparation steps to better illustrate effect sizes and variability. We hope these additions provide clearer and more transparent comparisons of workflow performance.  

**Reviewer Comment 3:** **Are the conclusions drawn adequately supported by the results?**

 The central operational conclusion HELP can achieve ARTIC-like coverage for many samples, especially at lower Ct / higher viral load and can maintain sequencing continuity during shortages is supported by the presented coverage and dropout data. Where conclusions drift beyond the data:

**1)** Claims about general robustness may be too broad given limited external validation (single lab setting, limited number of enzymes tested, limited replication described). 

**Response:** We appreciate the reviewer raising this point. Our intention was to describe robustness in the context of a drop-in alternative to the ARTIC LoCost workflow, where experimental conditions were kept consistent to enable direct comparison. We agree that the term should be interpreted within the scope of the conditions evaluated in this study, however we recognise that validation of the approach requires the user community to further validate the protocol, beyond the scope of the current study.

**We have therefore revised the manuscript to clarify that HELP demonstrated robust performance under the conditions tested**. In the Abstract: instead of  “ *ARTIC HELP provides a robust and flexible solution to maintain viral sequencing capacity when standard reagents are inaccessible or unaffordable*” we now use the following wording “ *ARTIC HELP provides a practical and flexible solution to maintain viral sequencing capacity when standard ARTIC LoCost reagents are inaccessible or unaffordable*”. (Conclusion; *lines 603-604*) Instead of “ *We developed ARTIC HELP, a robust workflow using alternative enzymes and home-brew buffers that works as well as the standard ARTIC LoCost protocol for sequencing viral genomes from clinical samples*” we now use the following wording “ *We developed ARTIC HELP, a workflow using alternative enzymes and home-brew buffers that performed comparably to the standard ARTIC LoCost protocol for sequencing viral genomes from clinical samples*.”

**2)** The cost inequity discussion is important but the inference that disparities are driven “rather than protocol complexity” is not fully demonstrated complexity is not quantitatively measured and procurement ecosystems differ. The table shows price variation the causal attribution should be softened or better supported. 

**Response:** We appreciate this important point. We agree that our analysis shows cross-country variation in reagent pricing but does not directly quantify the contribution of importation protocol complexity. We have therefore revised the manuscript to soften the causal interpretation and to state more cautiously that the observed disparities are likely influenced by import fees, shipping costs, taxes, and local procurement conditions. (Abstract) : Instead of *“..driven by import fees and supply barriers, rather than protocol complexity..*” we now use the following wording *“.. likely influenced by import fees, supply barriers, and local procurement conditions.*.” (Discussion, *lines: 578-581*) Instead of *“..with these cost increases arising not due to technical differences in the supplied products, but largely to customs fees, high import taxes, and shipping costs.*” we now use the following wording *“..with these cost differences likely reflecting a combination of customs fees, import taxes, shipping costs, and local procurement conditions*.”

**3)** Norovirus conclusions should be framed carefully: the data strongly suggest genotype/primer-scheme effects dominate coverage for some genotypes but additional experiments (primer mismatch analysis, rebalancing, alternate schemes) would be needed before recommending HELP broadly for “Norovirus GII” without caveats. 

**Response:** We are thankful for this constructive suggestion. We agree that the norovirus findings should be interpreted more cautiously. We have revised the manuscript to clarify that the results demonstrate the applicability of the HELP workflow for the genotypes tested, while also highlighting that performance was influenced by genotype-specific primer-scheme effects. We also adjusted the Discussion and Limitation sections. (Results section, *lines 426-429*) Instead of “ *These results demonstrated that the HELP workflow is robust and adaptable for sequencing Norovirus genogroup II, confirming its applicability beyond SARS-CoV-2*.” we now use the following wording  “ *These results indicate that the HELP workflow can be applied to Norovirus GII for the genotypes tested in this study, although performance was influenced by genotype-specific primer-scheme effects.*” (Limitation section, *lines 597-601*) instead of “ *Although the current norovirus scheme is still under development, our findings provide insights for further optimization*”, we now use the following wording “ *As the current norovirus scheme is still under development, our findings should be interpreted as supporting initial use for the genotypes tested, while highlighting the need for further optimization*.”

# Reviewer report: Reviewer response for version 1

**DOI:** 10.21956/wellcomeopenres.27252.r135094

**Published:** 2025-11-22

**Authors:** Leonardo C. Caserta

**Affiliations:** 1 Cornell University, Ithaca, NY, USA

The study provides valuable insights into the flexibility of tiled amplicon whole-genome sequencing protocols, offering alternative and cost-effective reagents for library preparation.

 One important metric, however, was not included: the average read length per sample (or per workflow). Amplicon sizes can vary significantly depending on the enzymes used, which in turn affects the number of reads generated. This variability is especially pronounced when using long-read sequencing technologies.

 If this metric is not included in the analysis, it should at least be acknowledged as a limitation of the study.

Is the work clearly and accurately presented and does it cite the current literature?

Yes

If applicable, is the statistical analysis and its interpretation appropriate?

Yes

Are all the source data underlying the results available to ensure full reproducibility?

Yes

Is the study design appropriate and is the work technically sound?

Yes

Are the conclusions drawn adequately supported by the results?

Yes

Are sufficient details of methods and analysis provided to allow replication by others?

Yes

Reviewer Expertise:

Virology, diagnostics, molecular biology, sequencing, viral evolution

I confirm that I have read this submission and believe that I have an appropriate level of expertise to confirm that it is of an acceptable scientific standard, however I have significant reservations, as outlined above.

# Response

**Published:** 2026-04-11

**Authors:** Ganna Kovalenko

**Affiliations:** University of California Irvine, Irvine, California, USA

We sincerely appreciate the reviewer’s positive evaluation and helpful suggestion, which helped us further improve the manuscript.

**Reviewer Comment:** The study provides valuable insights into the flexibility of tiled amplicon whole-genome sequencing protocols, offering alternative and cost-effective reagents for library preparation.

 One important metric, however, was not included: the average read length per sample (or per workflow). Amplicon sizes can vary significantly depending on the enzymes used, which in turn affects the number of reads generated. This variability is especially pronounced when using long-read sequencing technologies.

 If this metric is not included in the analysis, it should at least be acknowledged as a limitation of the study.

**Response: **We sincerely thank the reviewer for this helpful suggestion. The median read length metric was already included in the sequencing quality control metrics reported in the Extended data Tables S4–S6 but was not mentioned in the main text. Following this comment, **we have now clearly highlighted this metric in the main text to make it easier for readers to identify.** *(lines 202-209):* *Performance metrics included amplicon post-PCR concentration, read count, N50, median read length, number and percentage of mapped reads, mean and average depth, genome coverage breadth, and amplicon dropouts (regions with ≤20× depth), detailed in Extended data Table S4 (Stages 1 and 2), Table S5 (Stage 3 SARS-CoV-2), and Table S6 (Stage 3 Norovirus GII). Across all experimental stages, median read lengths were highly consistent between ARTIC LoCost and HELP workflows (~525 bp for SARS-CoV-2 and ~1 kb for Norovirus GII), demonstrating that the use of alternative polymerases and library preparation reagents in the HELP protocol does not alter amplicon size distribution.*
